# Gait Analysis Using Wearable Sensors

**DOI:** 10.3390/s120202255

**Published:** 2012-02-16

**Authors:** Weijun Tao, Tao Liu, Rencheng Zheng, Hutian Feng

**Affiliations:** 1 School of Mechanical Engineering, Nanjing University of Science and Technology, 200 Xiaolingwei Street, Nanjing 210094, China; E-Mails: taoweijun01@163.com (W.T.); fenght@mail.njust.edu.cn (H.F.); 2 Department of Intelligent Mechanical Systems Engineering, Kochi University of Technology, 185 MIyanokuchi, Tosayamada-Cho, Kami-City, Kochi 782-8502, Japan; 3 Nakano Lab, Institute of Industrial Science, The University of Tokyo, 4-6-1 Komaba, Meguro-ku, Tokyo 153-8505, Japan; E-Mail: topzhengrencheng@hotmail.com

**Keywords:** gait analysis, wearable sensors, human kinematics, joint kinetics, electromyography, clinical application

## Abstract

Gait analysis using wearable sensors is an inexpensive, convenient, and efficient manner of providing useful information for multiple health-related applications. As a clinical tool applied in the rehabilitation and diagnosis of medical conditions and sport activities, gait analysis using wearable sensors shows great prospects. The current paper reviews available wearable sensors and ambulatory gait analysis methods based on the various wearable sensors. After an introduction of the gait phases, the principles and features of wearable sensors used in gait analysis are provided. The gait analysis methods based on wearable sensors is divided into gait kinematics, gait kinetics, and electromyography. Studies on the current methods are reviewed, and applications in sports, rehabilitation, and clinical diagnosis are summarized separately. With the development of sensor technology and the analysis method, gait analysis using wearable sensors is expected to play an increasingly important role in clinical applications.

## 1. Introduction

Gait analysis is the systematic study of human locomotion. This type of analysis involves the measurement, description, and assessment of quantities that characterize human locomotion [1]. Through gait analysis, the gait phase can be identified, the kinematic and kinetic parameters of human gait events can be determined, and musculoskeletal functions can be quantitatively evaluated. As a result, gait analysis has been employed in sports, rehabilitation, and health diagnostics. For instance, in some types of sports training, the method is applied to recognize the faults in athlete performances so that they can improve [2–6]. In orthopedics and rehabilitation, gait analysis is used to monitor the patient healing progress [7–9]. In health diagnostics, a method to discriminate between asymptomatic subjects and patients with medial knee osteoarthritis based on accelerometers and gyroscopes was investigated [10], and the ambulatory monitoring method for applications to Parkinson’s disease also has been developed [11,12]. In the field of biomedical engineering, gait analysis has been a fundamental method and assistive tool to characterize human locomotion. Gait analysis has attracted the interest of researchers and clinicians.

Research on gait analysis has been conducted since the late 19th century, and its widespread application in biomedical engineering began with the availability of video camera systems [13–17]. A standard gait analysis method based on the multi-camera motion capture system and force platform with the capability of measuring ground-reaction forces was successfully developed and applied in a number of gait laboratories [18,19]. However, this standard gait analysis requires specialized locomotion laboratories, expensive equipment, and lengthy set up and post-processing times. Moreover, limitations in terms of the moving area and gait cycles for the observed subject/patient have been observed. To mitigate these problems, an alternative gait analysis method based on wearable sensors, which are inexpensive and can be applied outside the laboratory environment, was studied and has shown great prospects in the recent two decades.

In gait analysis using wearable sensors, motion sensors are worn or attached to various parts of the patient’s body, such as the foot and waist. These sensors, which may be accelerometers, gyrosensors, force sensors, strain gauges, inclinometers, goniometers, and so on, can measure various characteristics of the human gait [20,21]. The movement signal recorded by these sensors can be used to perform the gait analysis. For example, the temporal characteristics of gait are collected and estimated from body-worn accelerometers and pressure sensors inside footwear, as reported in several papers [22–26]. Ng and Chizeck used goniometric measurements at the hip, knee, and ankle joints to detect five different gait phases [27]. Slavelberg and Forner-Cordero reported on estimates of the three-dimensional (3D) ground reaction forces (GRFs) from the insole based on foot pressure data [28,29]. With the development of motion-sensing technology, an increasing number of wearable sensors will be developed for gait analysis in the future. Gait analysis using wearable sensors will thus be widely used in the clinical field.

The primary purpose of the current paper is to review the current status of gait analysis technology based on wearable sensors. Section 2 introduces the gait phases of human walking and the working principles of wearable sensors, which are the basis of human gait analysis. Section 3 reviews the research method and primary results on gait analysis using wearable sensors. Section 4 provides a comprehensive outlook for the various applications of gait analysis based on wearable sensors. The conclusions to this paper are presented in Section 5.

## 2. Basis of Human Gait Analysis

### 2.1. Introduction of the Gait Phases

Generally, human walking is a periodic movement of the body segments and includes repetitive motions. To understand this periodic walking course better and easier, the gait phase must be used to describe an entire walking period. In the past, normal events were conventionally used as the critical actions of separated gait phases. However, this practice only proved to be appropriate for amputees and often failed to accommodate the gait deviations of patients impaired by paralysis or arthritis. For example, the onset of stance has customarily been called the heel strike [30,31]. However, the heel of a paralytic patient may never be in contact the ground or may do so significantly later in the gait cycle. Similarly, initial floor contact may be made by the entire foot (flat foot), rather than having forefoot contact, which occurs later, after a period of heel-only support. To avoid these difficulties and other areas of confusion, the Rancho Los Amigos gait analysis committee developed a generic terminology for the functional phases of gait [32].

**Figure 1.**
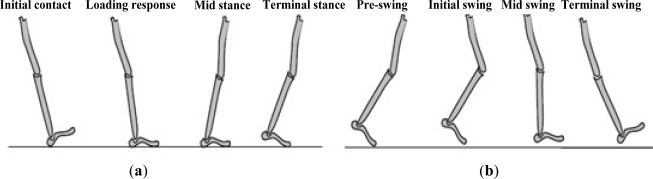
Gait phases in a normal gait cycle. (**a**) Gait phases of the stance period; (**b**) Gait phase of the swing period.

Analysis of the human walking pattern by phases more directly identifies the functional significance of the different motions generated at the individual joints and segments. In the present paper, a normal walking gait cycle is divided into eight different gait phases, that is, initial contact, loading response, midstance, terminal stance, pre-swing, initial swing, mid-swing, and terminal swing (Figure 1). Detailed definitions of the gait phases are described in the following [33,34].

1. Initial contact: This phase comprises the moment when the foot touches the floor. The joint postures presented at this time determine the limb’s loading response pattern.
2. Loading response: This phase is the initial double-stance period. The phase begins with initial floor contact and continues until the other foot is lifted for swing. Using the heel as a rocker, the knee is flexed for shock absorption. Ankle plantar flexion limits the heel rocker through forefoot contact with the floor.
3. Midstance: This phase is the first half of the single-limb support interval. In this phase, the limb advances over the stationary foot through ankle dorsiflexion (ankle rocker), while the knee and hip extend. Midstance begins when the other foot is lifted and continues until body weight is aligned over the forefoot.
4. Terminal stance: This phase completes the single-limb support. The stance begins with the heel rising and continues until the other foot strikes the ground, in which the heel rises and the limb advances over the forefoot rocker. Throughout this phase, body weight moves ahead of the forefoot.
5. Pre-swing: This final phase of stance is the second double-stance interval in the gait cycle. Pre-swing begins with the initial contact of the opposite limb and ends with the ipsilateral toe-off. The objective of this phase is to position the limb for swing.
6. Initial swing: This phase is approximately one-third of the swing period, beginning with a lift of the foot from the floor and ending when the swinging foot is opposite the stance foot. In this phase, the foot is lifted, and the limb is advanced by hip flexion and increased knee flexion.
7. Mid-swing: This phase begins as the swinging limb is opposite the stance limb and ends when the swinging limb is forward and the tibia is vertical (*i.e*., hip and keen flexion postures are equal). The knee is allowed to extend in response to gravity, while the ankle continues dorsiflexion to neural.
8. Terminal swing: This final phase of swing begins with a vertical tibia and ends when the foot strikes the floor. Limb advancement is completed as the leg (shank) moves ahead of the thigh. In this phase, limb advancement is completed through knee extension. The hip maintains its earlier flexion and the ankle remains dorsiflexed to neural.

Each gait phase has a functional objective and a critical pattern of selective synergistic motion to accomplish its goal. The sequential combination of the phases also enables the limb to accomplish three basic tasks, namely, weight acceptance, single-limb support, and limb advancement. Weight acceptance begins the stance period through initial contact and loading response. Single-limb support continues the stance through the midstance and terminal stance. Limb advancement begins in the pre-swing phase and continues through initial swing, mid-swing, and terminal swing.

Based on the above analysis of the gait phases and basic tasks of limb movement, the gait phases may be detected effectively after orientations of the leg segments are accurately obtained.

### 2.2. Wearable Sensors for Gait Analysis

Gait analysis has used different types of motion sensors and systems, such as the accelerometer, gyroscope, magnetoresistive sensors, flexible goniometer, electromagnetic tracking system (ETS), sensing fabric, force sensor, and sensors for electromyography (EMG). Based on these sensors, a single type or a combined sensor system of multiple types of sensors may be used for various gait analysis applications. The basic principles and features of these motion sensors and systems are described in the following.

#### 2.2.1. Accelerometer, Gyroscope, and Magnetoresistive Sensors

An accelerometer is a type of inertial sensor that can measure acceleration along its sensitive axis. The common operation principle of accelerometers is based on a mechanical sensing element that comprises a proof mass attached to a mechanical suspension system, with respect to a reference frame. The mass proof can be forced to deflect by the inertial force because of acceleration or gravity according to Newton’s second Law (force = mass × acceleration). Based on this principle, the acceleration can be measured electrically using the physical changes in the displacement of the proof mass, with respect to the reference frame.

Three common types of accelerometers are available, namely, piezoelectric, piezoresistive, and capacitive accelerometers [35]. Piezoresistive and capacitive accelerometers can provide dual acceleration components and have higher stability. Thus, these types of accelerometers are suitable for measuring the motion status in the human gait [36]. By attaching these accelerometers to the feet or legs, the acceleration/velocity of the feet or legs in the gait can be determined to perform the gait analysis [37].

A gyroscope is an angular velocity sensor. The micromachined gyroscope is based on the concept of measuring the Coriolis force, which is an apparent force proportional to the angular rate of rotation in a rotating reference frame. By detecting the linear motion from the Coriolis effort and performing an integration of the gyroscopic signal, the angular rate can be obtained. In addition, gyroscopes based on other operating principles also exist, such as the electronic, microchip-packaged MEMS gyroscope devices found in consumer electronic devices, solid-state ring lasers, fiber optic gyroscopes, and the extremely sensitive quantum gyroscope. A gyroscope can be applied for the measurement of the motion and posture of the human segment in gait analysis by measuring the angular rate [38–40]. For example, by attaching a gyroscope to human feet or legs, the angular velocity and angle of feet or legs during the gait can be determined to realize the reorganization of the various gait phases. In the gait analysis, a gyroscope is usually combined with an accelerometer to construct a complete initial sensing system.

**Figure 2.**
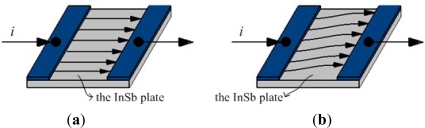
The model of magnetoresistive effect. (**a**) Current mode under non-magnetic field; (**b**) Current mode under magnetic field.

Magnetoresistive sensors are based on the magnetoresistive effect (Figure 2). If a magnetic flux (magnetic field) is not applied, the current flows straight through the InSb plate. However, if a magnetic flux is applied, a Lorentz force proportional to the magnetic flux density will deflect the current path.

As the current path is deflected, the current flows through the plate for a longer distance, causing the resistance to be increased. That is, the magnetoresistive effect refers to the change in the resistivity of a current carrying ferromagnetic material resulting from a magnetic field, with the resistance change proportional to the tilt angle in relation to the magnetic field direction [41]. Based on this magnetoresistive effect, magnetoresistive sensors can estimate changes in the orientation of a body segment in relation to the magnetic North or the vertical axis in the gait analysis [42–44]. Such sensors can provide information that cannot be determined by accelerometers or the integration of gyroscope signals.

#### 2.2.2. Flexible Goniometer

Unlike the inertial sensor, the flexible goniometer is operated by measuring the change in the physical signal resulting from the angular change. A flexible goniometer can be used to measure the relative rotation between two human body segments.

The flexible goniometers used in gait analysis can be divided into strain gauges, mechanical flexible, inductive, and optical fiber goniometers. A number of flexible electrogoniometers based on a strain gauge have been developed and used for angle measurement in gait analysis since the 1980s [45,46]. At present, numerous commercialized flexible electrogoniometers are available for the measurement of human posture and spinal motion [47–49]. A mechanical flexible goniometer is designed to obtain angular change by measuring the longitudinal displacement of two parallel wires bent in the plane of rotation, which is demonstrated by measuring the knee joint during human walking [50]. Laskoski *et al.* developed a goniometer with an inductive sensor for the measurement of human motion [51]. In addition, a type of optical fiber goniometer was developed recently and applied in human joint movement monitoring [52,53].

#### 2.2.3. Electromagnetic Tracking System (ETS)

The electromagnetic tracking system is a kind of 3D measurement device based on Faraday’s law of magnetic induction [54,55]. When an object carrying sensor coils performs a motion inside controlled magnetic fields, the induced voltages in the sensor coils will change, with respect to the change of the object’s position and orientation, relative to the source of controlled magnetic fields. In the ETS, the controlled magnetic fields are generated by a fixed transmitter and detected by the receivers fixed on the object in motion. Therefore, the positions and orientations of the object in relation to the transmitter can be calculated. Based on this working principle, some developed commercialized ETSs have been applied in bioengineering, including gait analysis [56,57] and the kinematic study of body segments [58–61].

#### 2.2.4. Sensing Fabric

The sensing fabric is a combination of sensing technology and fabric, which ranges from very superficially attached electronic components to a substitution of fibers and yarns with sensing properties inserted in normal fabrics, to electronic components made of fabric materials. Compared with other wearable sensors, the sensing fabric is more flexible and comfortable in measuring human posture and movement.

Generally, the sensing properties of the sensing fabric can be achieved by applying piezoresistive [62], piezoelectric [63], or piezocapacitive [64] materials, usually in the form of polymers, for their elastic properties. Different polymeric materials for use as coatings can be selected based on the sensing property desired for a particular application. In gait analysis research, printing carbon-based polymers onto stretchable fabrics, such as Lycra, can provide a perfect sensing fabric because of the compliant qualities and piezoresistive behavior of the polymer [65–67].

#### 2.2.5. Force Sensors

Force sensors can be embedded into footwear to realize ambulatory measurements of GRF during the gait. This GRF is a 3D vector, with the actual direction depending on the nature of the interface between the foot and the ground.

In the development of wearable force sensors, various implementations of the force transducer, including piezoelectrics [68,69], strain gauged [70,71] and capacitive transducerd [72–74], are feasible. In addition, Hessert *et al*. designed a type of wearable force sensor based on a photoelastic triaxial force transducer to measure GRF in gait analysis [75]. Force sensors based on the optical fiber matrix were developed to detect the shear and compressive force during human walking [76,77].

#### 2.2.6. Electromyography (EMG)

**Figure 3.**
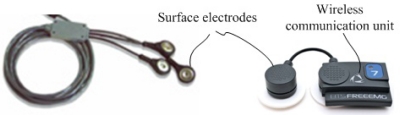
Commercialized wireless EMG sensors. The wireless EMG sensors include the electrodes and wireless communication unit.

To measure the action of the muscles in the lower extremity in a human gait, the EMG was developed to perform an indirect measurement of muscle activity using surface or wire electrodes. These electrodes are a kind of sensor for EMG and can detect voltage potentials to provide information on the timing and intensity of muscle contraction, which have been commercialized in combination with wireless technology as shown in Figure 3.

Generally, surface electrodes are used when only general information on muscle activity is required, whereas wire electrodes must be inserted into the designated muscle using a hypodermic needle to measure specific information on a particular muscle [78]. As a result, EMG sensors can be used to realize the assessment of muscle activity in human gait and play an important role in evaluating the walking performance of individuals with problems in their lower extremities [79–81].

#### 2.2.7. Commercialization of Wearable Sensors

Along with the wide application of wearable sensors in gait analysis, some commercialized wearable sensors have begun to appear. For example, a combination sensor system provided by Xsens Motion Technologies (Enschede, The Netherlands) has been proposed, which includes six degrees of freedom force and moment sensors and miniature inertial sensors to estimate joint moments and powers of the ankle [82]. The 3D displacements of center of mass during gait were calculated using measurements of the sensor system [83]. These two measurement systems were based on commercially available inertial measurement unite (IMU) or multi-axial force senor, and different algorithms were developed for the varied applications. A more compact system including all the 3D motion sensors and GRF sensors in shoes must be helpful for clinic gait analysis and the embedded control system of human assistant mechanism. The novel sensor system named M3D has been commercialized by TEC GIHAN Co. (Kyoto, Japan). In addition, A commercialized Motion Capture System named Polhemus Liberty ETS system (Polhemus Incorporated, Colchester, VT, USA) was also used to obtain the 3D gait kinematics by Mills *et al.* [57], and various commercialized EMG sensors were developed for the gait analysis, such as the Surface EMG Sensors of Delsys Incorporated (Boston, MA, USA) and the SX230 EMG Sensor of Biometrics Ltd. (Newport, UK).

## 3. Gait Analysis Methods Based on Wearable Sensors

In the past two decades, a great deal of attention has been given to gait analysis using wearable sensors, and significant progress has been achieved in this area. The achievements of human gait analysis can be divided into three areas, namely, kinematics, kinetics, and EMG. The kinematics of the human gait describes the movements of the major joints and components of the lower extremity in the human gait. Gait kinetics focuses on the study of forces and moments that result in the movement of human segments, in which the orientation of all the leg segments obtained from gait kinematics is often required. The EMG of the human gait is used to detect and analyze muscle activity during human walking. In this chapter, gait analysis methods based on wearable sensors are reviewed separately based on these three research areas.

### 3.1. Gait Kinematics

For gait analysis and its application in biomedical engineering, gait kinematics must be established on the basis of kinematic measurement and analysis. Kinematic measurement collects gait data using various sensors. Based on these collected gait data, a kinematic analysis can be performed to recognize the gait phases, as well as obtain the general gait parameters and movement information on the body segments. As a basis of gait kinematics, kinematic measurement is the essential principle that can significantly affect the selection of the kinematic analysis method.

Two main techniques are employed in the kinematic measurement of the human gait. One earlier developed technique is based on camera systems, which are used in a large number of specialized laboratories. The other technique for kinematic measurement is the use of wearable sensors, which was first proposed by Morris in 1973 and has been substantially developed as an ambulatory gait analysis method in the last two decades [84]. Among the two techniques, the latter represents the direction of future development and is the emphasis of the discussion in the current section.

The application of the accelerometer marked the beginning of gait analysis using wearable sensors and provides an effective method for analyzing human gait in ambulatory circumstances. Mathie *et al.* [85,86] reviewed the use of accelerometer-based systems in human movement, such as monitoring a range of different movements, measuring physical activity levels and identifying and classify movements performed by subjects, and discussed a real-time human movement classifier using a triaxial accelerometer for ambulatory monitoring. Godfrey *et al.* [87] gave a comprehensive comparison of all the achieved result on using accelerometers for human movement and mobility analysis from 1991 to 2006, summarized the various interpretation methods of accelerometer data and commercially available monitors based on the accelerometers. Moreover, Preece *et al.* [88] reviewed of the techniques which have been used to classify the normal and identify falls from body-worn sensors including accelerometers, gyroscope and other sensors for gait analysis. In some studies, accelerometers were mounted on the thigh and shank to measure accelerations during walking [89,90]. By analyzing these obtained acceleration data, the stages of lower extremity movement were determined [91]. The gait cycle time and number of walking steps can be determined using several accelerometers affixed to the subject’s waist [92]. In addition, the suitability of the integrated accelerometer to measure dynamic acceleration for gait applications was assessed and validated through a comparison of the predicted pendulum acceleration (derived from a goniometer) with the acceleration measured by the integrated accelerometer [93]. Various feature extraction methods for the classification of dynamic activities from accelerometer data were compared based on two datasets of activities collected from 20 subjects [94].

**Figure 4.**
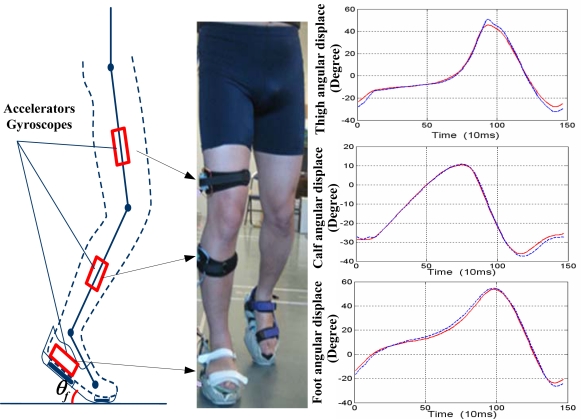
Kinematic measurement based on accelerators and gyroscopes. By using three pairs of accelerators and gyroscopes attached on the foot, calf and thigh seperately, the angular displacements of the lower extremity were measured.

To obtain more information on the human gait, other wearable sensors, such as gyroscopes and magnetoresistive sensors, are combined with accelerometers and applied in gait kinematics. Gyroscopes are usually applied in the measurement of angular rate and the angle of various joints on the lower extremities [95–97]. Such devices could be mounted on the foot, shank, or thigh to measure the angular rate and obtain angular information on various joints [98,99]. As shown in Figure 4, a kinematic measurement based on accelerators and gyroscopes was performed. In this measurement, the foot angular displacement, calf angular displacement and thigh angle (represented by the red lines) of the right lower extremity in a walking cycle were measured and compared with the results (represented by the blue lines) based on multi-camera system.

The magnetoresistive sensor can provide a reference measure for body orientation, in addition to the earth gravity field. Moreover, its sensor signal is unaffected by dynamic motions. Therefore, such a device is suitable for determining and estimating changes in the orientation of the lower extremity segment relative to the magnetic North or the vertical axis in the gait analysis.

In addition, the flexible goniometer, ETS, and sensing fabric also can serve as wearable sensors in gait analysis. Direct measurements of joint angles can be conducted through flexible goniometers, which have been used in clinical research [100,101]. ETSs can directly measure the 3D position and orientation of body segments in the human gait [102,103]. The primary limitations of ETSs are their limited capture volume and susceptibility to magnetic interference from metal objects within and near their capture volume. When these limitations are addressed, the accuracy of position and orientation data acquired from ETSs is comparable with that from image-based systems [104,105]. The smart sensing fabric can be easily integrated into truly wearable, instrumented garments and can be used to record the movement of body segments in gait kinematics because of their flexibility and conformability [106]. In the paper of Preece *et al.* [107], an automatic identification of gait events using an instrumented sock with piezo-resistive strain sensors was investigated.

Based on the measured data, detection of the gait phase and identification of the characteristics of human gait can be achieved through a variety of analysis techniques. In gait detection, various approaches have been applied to determine the gait events during the human walk, as reviewed by Rueterbories *et al.* [108]. Functional analysis, based on mathematical methods for curve sketching to extract features, is a common approach used to indicate certain gait phases or events [109,110]. Inductive machine learning, such as neural networks [91,111], mutual information classifiers [112], fuzzy computational algorithms [113,114], and support vector machines [115], have also been applied in gait detection using wearable sensors. In the identification of the characteristics of human gait, miniature accelerometers and gyroscopes have been used in estimating body segment postures and orientations based on a variety of algorithms. However, unknown initial joint angles and error accumulation in the integral value of the gyroscope is a significant problem.

To mitigate this problem, several studies have proposed and applied a variety of algorithms. For example, Cikajlo *et al*. [116] applied a Kalman filter to correct the shank inclination measured by a gyroscope. To incorporate the excellent dynamics of a gyroscope and the stable, drift-free performance of an accelerometer, a Kalman-based fusion algorithm was applied to obtain dynamic orientations of body segments [117]. Takeda *et al*. proposed a new method to visualize 3D gait using a stick-chain model and segment orientations estimated from measurements of accelerometers and gyroscopes in a global coordinate system [118]. In addition, the extended Kalman filter [119] and Gaussian particle filter [120] were also used to evaluate the hip angle in a walking cycle from the measurements of the wearable sensors, thus improving accuracy. According to the results of the kinematic analysis, the information on segment acceleration and velocity, joint angle, and gait events, such as heel strike and toe-off, can be provided and used in clinical applications.

### 3.2. Gait Kinetics

Gait kinetics is the study of forces and moments that result in the movement of body segments in a human gait, including the measurement of GRF and kinetic analysis. Kinetic measurement has largely focused on the forces between the foot and the ground, which depend on the application of adaptive force sensors. In kinetic analysis, proper and efficient calculation of the required joint moment of lower extremities during gait is the most basic and important task. Gait kinetics has been an important part of healthcare evaluation and the clinical diagnosis of body movement abilities in daily activities.

A number of studies [121–124] have reported that numerous stationary systems, such as force plates and instrumented treadmill devices, that are available for the measurement of GRF. Earlier methods for measuring GRF were usually based on the application of a stationary force plate fixed with a number of force sensors. However, a stationary force plate cannot measure more than one stride. Thus, in studies of continuous walking, a complex system comprising a large number of force plates and a data fusion method must be constructed [121,122]. An instrumented treadmill, which is a dynamometric platform formed by laying two force plates under a treadmill, can overcome some limitations of the system using distributed multiple force plates in successive measurements of the GRF for gait evaluation [123,124]. However, constraints would still be present because subjects would have to walk along a straight line and cannot realize turning or direction changes. In conclusion, such constraints imposed by GRF measurement based on stationary systems do not make the method feasible for measurements in everyday situations.

**Figure 5.**
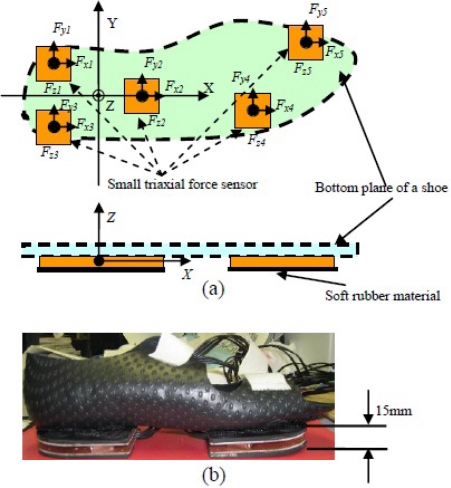
A wearable GRF sensor system constructed using five small triaxial force sensors. (**a**) Definition of sensors’ ordinate system and sensor mechanism; (**b**) Prototype of an instrumented shoe for right foot.

To overcome the limitations of stationary devices in GRF measurement, numerous studies have developed wearable sensors attached to shoes [125–128]. Pressure sensors have been widely used to measure gait and the distributed vertical component of GRF, as well as to analyze the loading pattern on the plantar soft tissue during the stance phase of gait [125,126]. However, in these systems, the transverse components of GRF (friction forces), which are some of the primary factors resulting in falling, have been neglected. By fixing two externally mounted sensors beneath the front and rear boards of a special shoe, an instrumented shoe for ambulatory measurements of center of pressure (CoP) and triaxial GRF in successive walking trials has been developed [127,128]. Our current research also developed a new wearable GRF sensor system, which has a thin and light sole and can be fixed on a pair of shoes to measure GRF [24]. As shown in Figure 5, the proposed wearable GRF sensor system was constructed using five small triaxial force sensors (USL06-H5-500N-C, weight: 15 g, size: 20 mm × 20 mm × 5 mm) made by TEC GIHAN CO., Japan.

**Figure 6.**
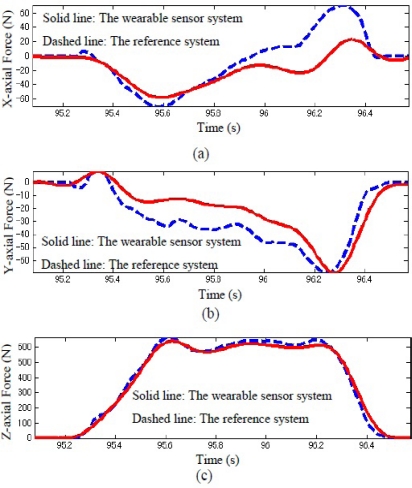
Triaxial GRF measured by the wearable sensor system (solid line) and referenced system (dashed line) during walking trial. (**a**) Comparison results of x-axial force (anterior-posterior direction); (**b**) Comparison results of y-axial force (medio-lateral direction); (**c**) Comparison results of z-axial force (vertical direction).

By mounting the five triaxial sensors on an aluminum plate beneath the shoe, all five local coordinate systems defined for each triaxial sensor could be aligned accurately with the global coordinates. Here, (*i* = 1, 2, 3, 4, and 5) indicate the triaxial forces measured by the distributed five triaxial sensors, and (*x_i_*, *y_i_*) is defined as the position of each triaxial sensor. The three components of GRF measured by the developed sensor system were obtained and compared with the measurement result using the reference sensor system in a representative walking trial, as shown in Figure 6. GRF and CoP can be easily calculated using the measured triaxial forces, *F_xi_*, *F_yi_*, and *F_zi_*, from the five small triaxial force sensors.

Based on the orientations of all the leg segments obtained from the gait kinematics, the measured triaxial GRF, and CoP, the kinetic analysis can be performed to obtain additional kinetic information in the form of joint moments and joint powers of the lower extremities. In the course of kinetic analysis, the inverse dynamics method has been widely used for moment calculation because of its simplicity and ease of application [129,130]. As input parameters, kinematic information on the foot, combined with GRF, determines the necessary moments and forces from the ankle. Kinetic information obtained from the ankle is used for kinetic calculations in the shank. The procedure is repeated from shank to thigh and from thigh to hip. Mechanical power can be calculated from the obtained joint moment producing angular velocity, and the mechanical energy consumption during the human gait can be estimated [131].

In informed research on gait analysis, wearable sensors have been adopted to calculate the joint kinetic analysis. In a study by Schepers *et al*., an ambulatory wearable sensor system was used to assess foot and ankle dynamics, considering only the GRF factor and neglecting body inertial parameters [132]. In our research, a wearable sensor system with a GRF sensor and motion sensors of segments were used to analyze the kinetics of the ankle, knee, and hip joints [133,134]. The achieved joint kinetics can be used for clinical decisions and assist in medical diagnosis. For instance, joint kinetics, specifically joint moments and joint powers, is available as an additional tool in the assessment of normal and pathological gait [135]. Further research is necessary to standardize the methods for defining kinetic variables to develop a more reliable process of analyzing gait in the clinical setting.

### 3.3. EMG and Muscle Force

**Figure 7.**
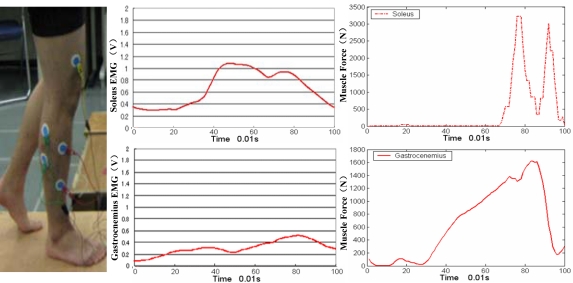
Diagram of EMG application in human gait. By using the EMG sensors, the electrical activity of muscles of lower extremity can be measured and the muscle force can be calculated.

Muscles provide power for human locomotion, and EMG is therefore used to detect and measure the small electric current produced by muscles during contraction. Wearable sensors can be placed on the skin to perform these measurements, as shown in Figure 7. EMG was developed during the first half of the 20th century, and the earliest major studies on EMG during walking were performed in the 1940s and 1950s [136]. Subsequently, considerable improvements in procedures and equipment have been achieved. The measurement of EMG in human walking is now an important method in clinical gait analysis. With the development of wireless technology and its application on sensors, EMG has become a very reliable and wearable tool for gait analysis.

**Figure 8.**
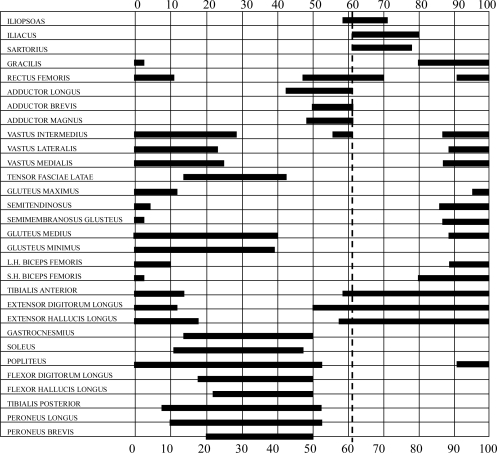
“On-off” diagrams of normal EMG activities during gait modification.

Numerous studies have investigated the electrical activity responsible for the phasic contribution of muscles in a gait cycle [137]. The phasic EMG activity of individual muscles depend on not only walking speed, age, and body size, but also on a number of technical issues involved in EMG collection. The magnitude of EMG signals may not be directly proportional to the tension created in a muscle with the changing phasic EMG. Thus, a number of studies illustrate EMG activity as simple “on-off” diagrams, as shown in Figure 8 [138]. Other studies attempt to provide more information through the normalization of the EMG linear envelope and the timing of functional muscle activity with peak activity, usually to the standard of EMG activity during maximal isometric contraction [139]. Estimates of phasic muscle activity and 3D motions of the limbs have been combined pictorially to provide an overall view of gait and muscle action [138]. Significant variability in the reported data is attributable to the sensitivity of muscle phasic contractile patterns to walking velocity, but evidence also show that normal physiologic walking strategies include some variation in stride to stride muscle timing. Depending on the presence or absence of neuromuscular disease, and on the specific muscle, EMG data from five to ten gait cycles may have to be averaged to obtain a representative sample.

EMG of the active muscle can yield information on muscle physiology and motor control beyond issues of timing during gait. Modern diagnostic EMG can identify neural injury or compression, denervated muscles, or primary pathological processes [140,141]. In particular, the ability to use EMG analysis to quantitative localized muscle fatigue has significant importance in the study of exercise physiology, athletic training, ergonomics, physical therapy, and physical medicines [142].

On the other hand, the EMG-to-force processing approach has also become a reliable technique for the estimation of muscle force [143–145], which belongs to the field of muscle kinetics. In this field, several studies proposed the static optimization method to calculate muscle force, which could obtain more accurate muscle force results than the EMG-to-force processing approach [146]. This research topic results in another important development for gait analysis, which is the biomechanics of muscles based on the musculoskeletal modeling method. In human gait, muscles of the lower extremity produce movement of the joint by generating linear force at a distance from the center of the joint. The farther the tendon of a muscle lies from the joint center, the greater the moment to produce movement will be [147]. The static and dynamic optimization solution is a converse method to estimate muscle force for gait analysis [148]. Visualization of a muscular skeletal system of the lower extremity in gait analysis becomes possible using two excellent software packages, namely, OpenSim and Anybody, separately [149,150].

## 4. Application of Gait Analysis Using Wearable Sensors

With the development of sensor technology and gait data analyzing techniques, gait analysis using wearable sensors has become a widespread and useful tool for both clinical practice and biomechanical research. Using small, low-power, and low cost wearable sensors, ambulatory gait analysis can be used conveniently in sports, rehabilitation, and clinical diagnostics, as summarized in the following.

### 4.1. Sports

In sports, gait analysis based on wearable sensors can be used for sport training and analysis and for the improvement of athlete performance. An athlete’s faulty performance can be recognized and further corrected by the ambulatory gait analysis, which can promote performance improvement. A number of studies have applied the ambulatory gait analysis technique to analyze the movement of body segments in various sporting events, including golf [151], running [152], and baseball training [153]. The gait analysis technique based on wearable wireless sensors has also been used in exercise coaching [154] and mobile interactive entertainment driven by exercise [155]. The prevention of sport injury is an alternative application of gait analysis using wearable sensors. Combining gait analysis with sport training can effectively prevent many injuries from overuse or incorrect posture and motion, thus maintaining the athletes’ high level of running and jumping [152].

### 4.2. Rehabilitation

The application of gait analysis in rehabilitation has been widely studied and realized in numerous hospitals and healthcare centers with subjects of different ages. Gait analysis based on wearable sensors is an effective clinical tool for treatment planning, outcome assessment, and longitudinal studies on maintenance and progress.

As a clinical tool, motion analysis of the lower extremities during gait is applied in pre-operative planning for patients with cerebral palsy [156–158] and can alter surgical decision making. After the application of gait analysis, the cost of care can be reduced by decreasing the number of pre- and post-operative clinic visits and subsequent surgical or other interventions. Ambulatory gait analysis can also provide a quantitative description of the gait cycle, which complements and augments the standard observational analysis. The ambulatory gait analysis results can also assist in interventions to determine whether or not a particular course of treatment is appropriate for a patient. For example, in such surgical procedures as the rectus femoris transfer, analyzing the swing phase of a patient’s gait can indicate the necessity of undergoing the procedure [159].

For people with neurological conditions, such as Parkinson’s disease and stroke, the ambulatory gait analysis is an important step in their recovery process and can provide low-cost and convenient rehabilitation monitoring. Parkinson’s disease is commonly characterized by motor dysfunctions, such as resting tremors, slowing of movement, gait difficulty, and limb rigidity. Hence, gait has been verified as one of the most reliable diagnostic signs of this disease. Accordingly, studies on the use of gait analysis as an alternative measure of the severity of Parkinson’s disease have been increasing. Salarian *et al*. [160] performed gait measurement in patients with Parkinson’s disease using a developed wearable sensor device and concluded that stride length is highly correlated with the severity of the disease. Furthermore, gait phase discrimination [161] and gait monitoring methods [162] based on the wearable sensor system have been developed for patients with Parkinson’s disease. The assessment of the freezing-of-gait symptom and the gait-unfreezing method [163,164] has also been established using the wearable assistant. In the rehabilitation of stroke patients, gait analysis using wearable sensors also play an important role. Many stroke patients who regained their walking ability do not have sufficient locomotion capacity for independent mobility in their community and need to undergo gait rehabilitation for the recovery of their independent mobility. Therefore, ambulatory gait analysis is applied in the automatic identification of temporal gait parameters of post-stroke individuals to provide an assessment of the functional utilization of the affected lower extremity as part of the behavior enhancing feedback [165,166]. In recent years, the application of gait analysis based on wearable sensors has made significant progress and achieved great success in the rehabilitation of post-stroke individuals [167,168].

In the field of joint arthroplasty, clinical and instrumental data can be obtained through gait analysis based on wearable sensors. Such data can be used to evaluate the patient’s progress before and after hip or knee arthroplasty. Aminian and Najafi [169] tested and validated the application of gait analysis based on body-fixed sensors in hip osteoarthritic patients as a progress assessment method before and after surgery. Accurate assessment of the progressive improvement in gait following knee arthroplasty based on the ambulatory gait analysis was proposed, and the effectiveness of this method was also validated [170]. In addition, the EMG technique can be used to determine the intensity of muscle contraction and evaluate the walking performance of individuals with neurological injuries during rehabilitation [171].

### 4.3. Clinical Diagnosis and Healthcare Monitoring

In the clinical diagnosis of patients with Parkinson’s or knee osteoarthritis disease, the ambulatory estimation of lower extremity movement in the gait is usually necessary [172]. Based on the estimation results of the lower extremities, the disease and its severity can be determined, and clinicians can establish a proper treatment scheme for the patients. In healthcare monitoring, gait analysis based on wearable sensors can also be applied in various occasions, such as in the detection of gait abnormalities, the assessment of recovery, fall risk estimation, and so on. In the healthcare environment, gait information is used to detect walking behavior abnormalities that may indicate the onset of adverse health problems or the progression of neurodegenerative diseases [173]. The presence of gait abnormalities in elderly persons is often a significant predictor of the risk of the development of dementia, especially non-Alzheimer’s dementia [174].

Fall risk estimation is also an important application of gait analysis using wearable sensors. As the most common type of home accident among elderly people, fall is a major threat to health and independence. The importance of this threat facilitated studies on fall risk estimation to provide adaptive assistance and preventive measures to subjects deemed at risk. Nyberg *et al*. analyzed and reported the reasons for falling and the phase in which fall occurs based on gait analysis using wearable sensors [175,176]. Moreover, a number of studies developed different algorithms to judge the existence of an emergency fall [177–182]. Bourke *et al.* developed a threshold-based fall-detection algorithm using bi-an axial gyroscope sensor, and investigated the fall-detection algorithms using a waist-worn tri-axial accelerometer or a custom vest embedded with a tri-axial accelerometer [177–179]. Kanges *et al.* determined the acceleration thresholds for fall detection using tri-axial accelerometers attached at the waist and head, analyzed the sensitivity and specificity of fall detection in people aged 40 years and over, and gave an overall comparison of different fall-detection algorithms [180–182]. These research results showed that the wearable sensors for gait analysis can be applied to the fall-detection and fall risk estimation in daily life. As a result, this technique can be combined with wireless network and play an important role in smart home technology, which aims to support the ability of older adults and people with disabilities to remain living independently in their own homes and provide appropriate life assistance and health monitoring in their daily life.

## 5. Conclusions

A useful tool both in clinical practice and biomechanical research, gait analysis has attracted an increasing amount of attention from the researchers and clinicians since the 1970s. With the utilization of video cameras, gait analysis based on highly accurate computer-based force plates was established in the 1980s and was applied in specialized motion laboratories. Subsequently, gait analysis using wearable sensors provided quantitative and repeatable results over extended time periods with low cost and good portability, showing better prospects and making great progress in recent years. At present, commercialized wearable sensors have been adopted in various applications of gait analysis.

The current paper provides a systemic review of gait analysis using wearable sensors. After the introduction of the human gait phase, a number of wearable sensors used in gait analysis were described, and their basic principles and application features were identified, providing the basis for ambulatory gait analysis. Studies on gait analysis methods based on wearable sensors, which are divided into gait kinematics, gait kinetics and EMG, was reviewed according to the detailed research aim. Furthermore, the applications of these methods in sports, rehabilitation, and clinical diagnosis were covered individually. Significant application prospects of the proposed methods can be expected and exploited.

Until now, gait analysis using wearable sensors has made great progress and shown good application prospects. In order to realize the wide application of this technology in our life, some detailed technical matters still need to be improved, such as the stability of sensor signals, the reliability of analytical algorithms for kinematics and kinetics in gait analysis, development of low cost and small volume integrated wearable sensor systems, and so on. In the future, the intelligent gait analysis system, which can perform the gait kinematics, kinetics and EMG simultaneously by the integration of multiple wearable sensors with small volumes, low power, and wireless data transfer, will be an important development trend. Another important trend is the fusion of gait analysis technology and remote medical systems, whereby gait analysis results can be transmitted to a centralized medical location and processed by trained medical personnel. Once these two developments are achieved, gait analysis using wearable sensors will be widely performed in daily environments, various clinical occasions, and other possible applications to monitor physical activity. In the prospective smart home, gait analysis technology based on wearable sensors also can play an important role as a medical assistance tool.
